# Weekly full-dose gemcitabine and single-dose cisplatin with concurrent radiotherapy in patients with locally advanced pancreatic cancer

**DOI:** 10.1038/sj.bjc.6604247

**Published:** 2008-02-26

**Authors:** S P Hong, J Y Park, T J Jeon, S Bang, S W Park, J B Chung, M-S Park, J Seong, W J Lee, S Y Song

**Affiliations:** 1 Division of Gastroenterology, Department of Internal Medicine, Yonsei Institute of Gastroenterology, Yonsei University College of Medicine, Seoul, Korea; 2 Department of Diagnostic Radiology, Yonsei Institute of Gastroenterology, Yonsei University College of Medicine, Seoul, Korea; 3 Department of Radiation Oncology, Yonsei Institute of Gastroenterology, Yonsei University College of Medicine, Seoul, Korea; 4 Department of Surgery, Yonsei Institute of Gastroenterology, Yonsei University College of Medicine, Seoul, Korea; 5 Brain Korea 21 Project for Medical Science, Yonsei University College of Medicine, Seoul, Korea

**Keywords:** pancreatic cancer, gemcitabine, cisplatin, concurrent chemoradiotherapy

## Abstract

The aim of this study was to evaluate the efficacy and the toxicity of a full dose of gemcitabine and a single dose of cisplatin with concurrent radiotherapy in patients with locally advanced pancreatic cancer. Forty-one patients with locally advanced pancreatic cancer were enrolled. Patients received gemcitabine (1000 mg m^−2^ on days 1, 8, 15, 29, and 36) and cisplatin (70 mg m^−2^ on days 1 and 29) with concurrent radiotherapy (45 Gy in 25 fractions). Treatment was completed in 38 out of 41 patients (92.7%). The overall response rate was 24.4% (two complete and eight partial). Six patients (14.6%) underwent definite pancreatic resection and four had negative surgical margins. The intention of the treatment analysis showed that the median survival time and median time to tumour progression were 16.7 and 8.9 months. The 1- and 2-year survival rates were 63.3 and 27.9%, respectively. Overall survival was significantly longer in the low baseline CA19-9 group and therapeutic responders. Toxicities were tolerable and successfully managed by conservative treatments. The therapeutic scheme of a weekly full dose of gemcitabine and a single dose of cisplatin combined with external radiation is effective and might prolong the survival of patients with locally advanced pancreatic cancer.

Pancreatic cancer is a leading cause of cancer-related mortality in western countries and accounts for approximately 30 000 deaths each year in the United States (Jemal *et al*, 2005). In Korea, pancreatic cancer accounts for the eighth highest cancer incidence and the fifth highest cancer-related mortality (Ministry of Health and Welfare Republic of Korea, 2007). Surgical resection is the only potentially curative therapy, but only 10–20% of patients qualify for the procedure (Kalser *et al*, 1985). The invasion of pancreatic cancer into the major vessels is the main reason why resections cannot be performed. Approximately 30% of patients are diagnosed with locally advanced disease at initial presentation (Moertel *et al*, 1981). In this group of patients, there are several reports of favourable results with concurrent chemoradiotherapy.

Gastrointestinal Tumor Study Group (1979, 1988) (GITSG) trials demonstrated a survival benefit for patients with locally advanced pancreatic cancer who were treated by external beam radiotherapy and 5-fluorouracil (5-FU) compared with patients who were treated by radiotherapy alone (Moertel *et al*, 1981). In recent decades, 5-FU has been considered the standard cytotoxic agent and radiosensitiser for use with concurrent radiotherapy. Since 1996, gemcitabine, a pyrimidine analogue and potent radiosensitiser, has been studied as a substitute for 5-FU in treatments using concurrent chemoradiotherapy (Blackstock *et al*, 1999; McGinn *et al*, 2001). Recent phases I and II studies on gemcitabine-based chemoradiotherapy were feasible and improved therapeutic response and survival (Blackstock *et al*, 1999; McGinn *et al*, 2001; Okusaka *et al*, 2004; Magnino *et al*, 2005).

Many studies have been performed to increase the objective response rate (ORR) and overall survival (OS) by mixed regimens consisting of gemcitabine with other drugs. The combination of gemcitabine and cisplatin has a synergistic effect *in vitro* in which gemcitabine can inhibit the DNA repair mechanism after cisplatin-induced damage, and cisplatin influences gemcitabine metabolism through the inhibition of ribonucleotide reductase (Peters *et al*, 1995; Bergman *et al*, 1996). A recent phase III trial with gemcitabine (1000 mg m^−2^) and cisplatin (50 mg m^−2^) on days 1 and 15 of a 4-week cycle showed favourable median OS and median time to tumour progression (TTP) compared with gemcitabine alone in advanced pancreatic cancer (Heinemann *et al*, 2006). We recently reported the feasibility and clinical efficacy of cisplatin combined with weekly gemcitabine treatments for patients with metastatic pancreatic cancer (Bang *et al*, 2006b).

In the present study, we evaluated the efficacy and toxicity of concurrent chemoradiotherapy with a full dose of gemcitabine and a single dose of cisplatin in patients with locally advanced pancreatic cancer. The primary end point of this study was the survival rate, whereas the secondary end points were ORR, TTP, and side effects.

## MATERIALS AND METHODS

### Eligibility criteria

Patients were recruited from January 2000 to December 2005 at the Center for Gastrointestinal Diseases at Severance Hospital in Seoul, Korea. Seven inclusion criteria were used for enrollment: (1) patients must have a newly diagnosed and pathologically confirmed locally advanced pancreatic adenocarcinoma. We defined this cancer stage as when the tumour encases the superior mesenteric arteries or coeliac axis, or occludes the superior mesenteric–portal vein confluence. In these cases, we found no evidence of distant metastatic diseases based on physical examination and radiographic imaging, including positron emission tomography (PET) scans. The tumours were staged according to the American Joint Committee on Cancer (AJCC) staging system (6th edition). (2) Patients must be between the ages of 18–75 years. (3) Patients must also have an Eastern Cooperative Oncology Group (ECOG) performance status of 0–2; (4) adequate bone marrow function (white blood cell count ⩾4000 mm^−3^, absolute neutrophil count ⩾1500 mm^−3^, haemoglobin ⩾10 g per 100 ml, and platelet count ⩾100 000 mm^−3^); (5) adequate hepatic function (alanine transaminase or aspartate transaminase levels <5 times the upper limit of institutional normal and bilirubin levels <3 mg per 100 ml with adequate biliary decompression); (6) adequate renal function (serum creatinine <2 mg per 100 ml); and (7) adequate cardiopulmonary function. Patients were ineligible if they had a concurrent type of malignancy, experienced recent upper gastrointestinal bleeding, or any other underlying serious medical conditions that would interfere with the study. This study was approved by the Institutional Review Board of Severance Hospital. We fully informed all patients about the nature and purpose of the study and all patients gave written informed consent.

### Treatment plan

The protocol consisted of a 5-week course of external radiotherapy and concurrent chemotherapy with gemcitabine and cisplatin. Chemotherapy began on the 1st day of radiotherapy: 1000 mg m^−2^ of gemcitabine was delivered intravenously for 30 min on days 1, 8, 15, 29, and 36, while 70 mg m^−2^ of cisplatin was delivered intravenously for 2 h on days 1 and 29. Radiotherapy was administered by linear accelerator (CLINAC 2100C; Varian Medical Systems, Palo Alto, CA, USA) using three-dimensional conformal technique. A total radiation dose of 45 Gy was delivered with daily doses of 180 cGy for five fractions each week. Gross target volume (GTV) was confined to primary tumour and any regional lymph nodes over 10 mm detected by computed tomography (CT). Clinical target volume (CTV) was defined as GTV plus primary echelon lymph node according to tumour location. Planning target volume was defined as CTV plus 0.5 cm margin to cover organ motion and daily set-up error. The four parallel opposing fields (anterior, posterior, and opposed lateral fields) were used, and the radiation dose to the adjacent organs was limited as follows: liver ⩽30 Gy, kidneys ⩽20 Gy, and spinal cord ⩽45 Gy).

### Response assessments and maintenance therapy

The primary end point of this study was survival rate, and the secondary end points were ORR (complete response (CR)+partial response (PR)), TTP, and side effects. All patients underwent a pretreatment evaluation consisting of medical history, physical examination, laboratory tests including serum carbohydrate antigen (CA) 19-9, chest X-rays, and high-resolution pancreatic CT scans. These tests were performed within 2 weeks before the start of treatment. Positron emission tomography scans were performed to exclude distant metastases. Endoscopic retrograde cholangiopancreatography and biliary drainage procedures were performed if necessary.

Four weeks after completion of radiotherapy, therapeutic responses were evaluated according to the World Health Organization (WHO) criteria by using chest X-rays and helical CT scans (World Health Organization, 1979). A CR was defined as the disappearance of all measurable or evaluable diseases for a minimum of 4 weeks. Partial response was defined as a >50% reduction in the sum of the products of the perpendicular diameters of all measurable lesions for at least 4 weeks. SD was defined as a <50% reduction or a <25% increase in the sum of the products of the two perpendicular diameters of all measured lesions. Progressive disease (PD) was defined as a >25% increase in the sum of the products of the two perpendicular diameters of all lesions or the appearance of any new malignant disease. Patients who did not receive objective evaluations were regarded as PD. If the residual tumours became resectable after chemoradiotherapy, radical resections were performed. Patients with unresectable tumours received maintenance chemotherapy, which was gemcitabine (1000 mg m^−2^ weekly followed by 1 week of rest) and cisplatin (75 mg m^−2^ on day 1, every 4 weeks). Treatment with gemcitabine and cisplatin continued until there was evidence of disease progression or significant clinical deterioration. Follow-up evaluations were performed every third cycle. Time to tumour progression was calculated from the time of entry into the study until disease progression, and OS was calculated from study entry to death or the last follow-up visit.

### Toxicity and dose adjustment

During chemoradiotherapy, all patients visited the clinic every week and had blood samples taken every 2 weeks for toxicity evaluations. Toxicity was evaluated according to the National Cancer Institute Common Toxicity Criteria (NCI-CTC) version 3.0. The gemcitabine and cisplatin doses were reduced for toxicity on the day of treatment as follows: 75% of the drug dosage was given to patients with an absolute neutrophil count of 500–900 mm^−3^ or with a platelet count of 50 000–74 000 mm^−3^, and doses were reduced 50% if patients had an absolute neutrophil count of less than 500 mm^−3^ or a platelet count of 50 000 mm^−3^. For patients with WHO grades 3–4 nonhaematological toxicity (excluding nephrotoxicity), doses were reduced 75% for those with grade 3 nonhaematological toxicity (except nausea/vomiting and alopecia) and 50% for those with grade 4 nonhaematologic toxicity (except nausea/vomiting and alopecia). Radiation therapy was stopped when patients experienced grades 3–4 gastrointestinal toxicities or grade 4 haematological toxicities. Treatments were completely stopped after a second interruption caused by haematological toxicity.

### Statistical analysis

Objective responses were reported according to an intention-to-treat basis. On the basis of the most conservative assumption of a 30% survival rate at 1 year (null hypothesis) in historic controls with locally advanced pancreatic cancer (Okusaka *et al*, 2004), an increase of 50% or more (alternative hypothesis) could be shown with a power of 80% by investigating a sample size of at least 35 patients (*α*=0.05, one-sided test) (A'Hern, 2001). Overall survival and TTP were analysed using Kaplan–Meier method with 95% confidence intervals (CIs). The univariate analysis to identify parameters predicting survival was performed by computing survival curves using Kaplan–Meier method. A *P*-value of less than 0.05 was considered to be statistically significant. When using CA19-9 levels to predict survival, we excluded patients with serum bilirubin levels that were higher than 3 mg per 100 ml at the time when CA19-9 measurements were taken. All statistical analyses were performed with commercially available software (SPSS version 13.0; SPSS Inc., Chicago, IL, USA).

## RESULTS

### Patient characteristics

From January 2000 to December 2005, 41 patients were enrolled in the study. The baseline characteristics of patients are summarised in Table 1. The patient group had 22 men and 19 women with a median age of 59 years (range: 37–72 years). Fifteen patients (36.6%) showed obstructive jaundice at diagnosis and their median serum bilirubin level was 7.0 mg per 100 ml (range: 2.5–24.3 mg per 100 ml). Endoscopic biliary drainage with a plastic stent was performed on 13 patients while percutaneous biliary drainage was performed on 2 patients. Thirty-three patients (80.5%) had elevated CA19-9 levels (>37 U ml^−1^) upon initial diagnosis with the median CA19-9 level at 686 U ml^−1^ (range: 51–20 000 U ml^−1^). The median follow-up period was 15.6 months (range: 5.5–52.4 months).

### Objective responses, TTP, and OS

Of the 41 patients, 38 (92.7%) received scheduled chemoradiotherapy. Two patients could not complete the treatment schedule due to poor general conditions and one patient due to liver abscess. In all, 40 out of the 41 enrolled patients (97.6%) received an objective response evaluation and 1 patient was unable to be evaluated for response due to withdrawal from treatment before re-evaluation. This patient was considered to have PD.

Responses were analysed on an intention-to-treat basis. The ORR was 24.4% while CR and PR were achieved in two patients (4.9%) and eight patients (19.5%), respectively. Twenty-six patients (63.4%) and five patients (12.2%) showed SD and PD, respectively. Among the four out of five patients who can be evaluated for PD, two had lung metastasis and 2 had metastasis to the liver and multiple lymph nodes. When considering only the primary pancreas tumours, two patients had PR, and two patients had SD. Among the 20 patients who could be evaluated for serum CA19-9 levels, 4 (20%) had normalised CA19-9 levels and 12 (60%) achieved more than a 25% reduction in CA19-9 levels after chemoradiotherapy. After completion of chemoradiotherapy, six patients (14.6%) underwent surgery, four had R0 resections (margin negative), and two had R1 resections with positive margins. In all, 25 (83.3%) out of 30 patients without surgery received maintenance chemotherapy with a median of 3 cycles (range: 1–9 cycles).

**Figure 1:**
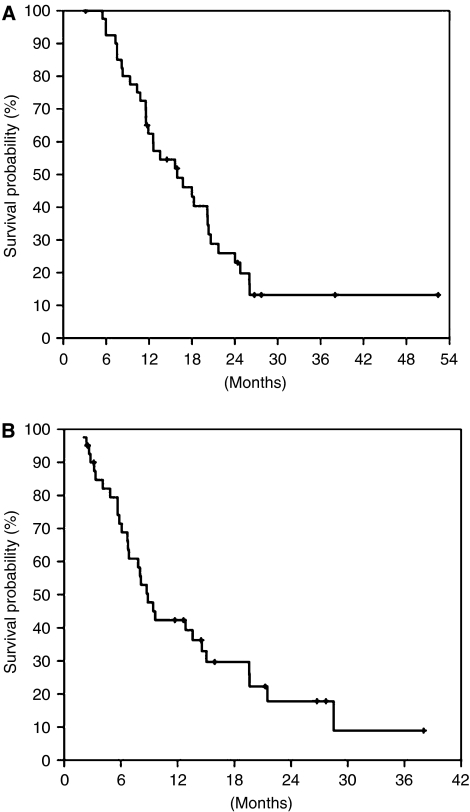
Overall survival (**A**) and TTP (**B**) of the enrolled 41 patients.

The median survival time of the 41 enrolled patients was 16.7 months (95% CI: 10.4–23.1 months) (Figure 1A). The median OS of resected and unresected patients was 21.7 months (95% CI: 14.1–29.3 months) and 12.6 months (95% CI: 7.5–17.7 months), respectively (*P*=0.079). The 1- and 2-year cumulative survival rates were 63.3 and 27.9%, respectively. The median TTP was 8.9 months (95% CI: 7.0–10.7 months) (Figure 1B).

### Survival predictor

**Figure 2:**
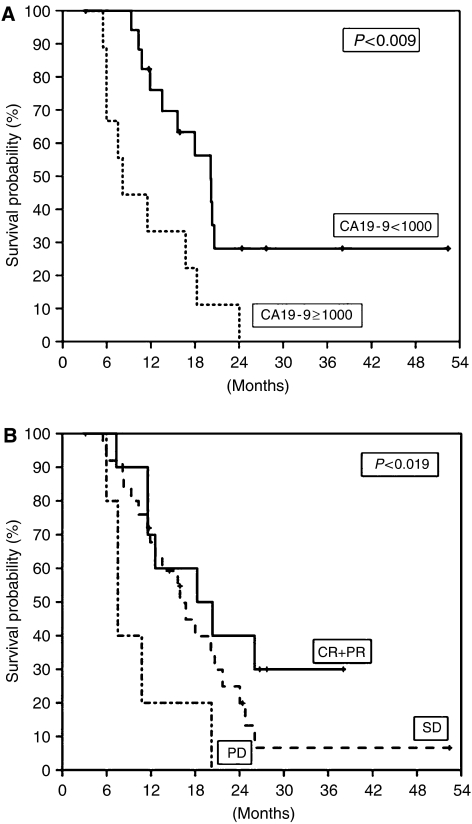
Cumulative survival of patients based on baseline CA19-9 level (**A**) and objective response (**B**). The median survival time of patients with baseline CA19-9 level less than 1000 U ml^−1^ and more than 1000 U ml^−1^ were 20.1 and 8.2 months, respectively. The median survival time of patients with objective response (CR+PR), SD, and PD were 18.3, 16.7, and 7.5 months, respectively.

The prognostic factors influencing the patients' cumulative survival were analysed (Table 2). Univariate analysis revealed four significant predictors of survival: performance status (*P*=0.002), baseline weight loss (*P*=0.022), baseline serum CA19-9 levels (*P*=0.004), and objective therapeutic response (*P*=0.019) (Figure 2).

### Toxicity

All 41 patients enrolled in the study were assessed for toxicity. Grades 3–4 toxicities included neutropaenia (26.9%), thrombocytopaenia (19.5%), nausea/vomiting (22%), diarrhoea (4.9%), and infection (2.4%) (Table 3). No treatment-related deaths occurred. Thirty-eight (92.7%) patients received full-dose radiotherapy (45 Gy), and three patients (7.3%) received a 75% reduced dose of gemcitabine and cisplatin due to myelosupression. One patient was hospitalised due to duodenal ulcer bleeding after completion of chemoradiotherapy.

## DISCUSSION

Chemoradiotherapy affects the local control of a main tumour and downstages the primary tumour by reducing the tumour size, nodal involvement, and vascular invasion. Primarily unresectable pancreatic cancer might converge into a resectable tumour after chemoradiotherapy, potentially allowing the patient to expect long-term survival after curative resection (Snady *et al*, 2000; Ammori *et al*, 2003). In a chemoradiotherapeutic setting, the chemotherapeutic agent should be a potent radiosensitiser to maximise the therapeutic gain of radiotherapy on primary tumours and have a systemic cytotoxicity to reduce the early metastasis of pancreatic cancer (Moertel *et al*, 1981; Blackstock *et al*, 1999).

In the present study, we used a combination of gemcitabine and cisplatin with concurrent radiotherapy. Although we found no consensus between administration schedules for gemcitabine with radiation, recent studies have used twice weekly or weekly administrations of gemcitabine for their main therapeutic schedules (Blackstock *et al*, 1999; McGinn *et al*, 2001; Okusaka *et al*, 2004; Magnino *et al*, 2005). According to the fact that patients with resectable pancreatic cancer developed early recurrence after surgery due to micrometastases and distant early metastases were important cause of treatment failure of chemoradiotherapy in patients with locally advanced pancreatic cancer, we used weekly gemcitabine treatments in our study (Yeo *et al*, 1997; Paulino and Latona, 2000). This schedule provided a modest local control and systemic cytotoxicity compared with biweekly administration, which was primarily focused on the effect of the radiosensitiser. In addition, we used a full dose of gemcitabine (1000 mg m^−2^) to maximise systemic cytotoxicity. Although most of the recent phases I and II studies were performed using a submaximal dose of gemcitabine with radiotherapy, a study by McGinn *et al* (2001) reported that the standard full dose of gemcitabine was acceptable. Our previous experience also showed that radiotherapy with a full dose of gemcitabine was tolerable (Chung *et al*, 2004).

Many attempts have been made to increase the ORR and OS by exploring the combination of gemcitabine with other chemotherapeutic agents. In this study, we used a single dose of cisplatin combined with gemcitabine. There is a report that median TTP and ORR were significantly improved with combined chemotherapy of gemcitabine and cisplatin in patients with locally advanced and metastatic pancreatic cancer (Colucci *et al*, 2002; Heinemann *et al*, 2006). Recent phases I and II trials of chemoradiotherapy with a combination of gemcitabine and cisplatin in locally advanced pancreatic cancer patients used a divided dose of cisplatin (Brunner *et al*, 2003; Wilkowski *et al*, 2004; Haddock *et al*, 2007). However, in a study by Shepherd *et al*, cisplatin was administered in a single dose over a 4-week cycle, and therapeutic effect was superior over divided dose administrations in patients with non-small cell lung cancer.

Previous phases I and II studies were performed with radiotherapy doses of 42–50.4 Gy and sometimes used boosting doses to achieve local control (Blackstock *et al*, 1999; McGinn *et al*, 2001; Brunner *et al*, 2003; Okusaka *et al*, 2004; Magnino *et al*, 2005). In the present study, we used radiation doses of 45 Gy with 25 fractions. Although four out of five patients with PD who could be evaluated had metastatic disease, two had PR and two had SD of the primary tumour after chemoradiotherapy. Our therapeutic schedule and regimen showed good local control and demonstrated that the treatment failure by concurrent chemoradiotherapy on locally advanced pancreatic cancer was caused not by local progression but early metastasis. These results are consistent with a previous study (Paulino and Latona, 2000).

We summarised recent phase II trials on chemoradiotherapy combined with gemcitabine alone or gemcitabine plus cisplatin in locally advanced pancreatic cancer (Table 4) (de Lange *et al*, 2002; Li *et al*, 2003; Okusaka *et al*, 2004; Wilkowski *et al*, 2004; Magnino *et al*, 2005; Haddock *et al*, 2007). Compared with the recent phase II study by Haddock *et al*, although the dose and cycle differed from our study, 24.4% ORR and 16.7 months median survival time of our study showed favourable results.

The major grades 3 and 4 toxicities were haematologic and gastrointestinal toxicities when gemcitabine was combined with radiotherapy. Rates of grades 3 and 4 toxicity in the present study were similar to other reports of gemcitabine-based chemoradiotherapy (Blackstock *et al*, 1999; McGinn *et al*, 2001; Brunner *et al*, 2003; Okusaka *et al*, 2004; Magnino *et al*, 2005). In the present study, grades 3 and 4 neutropaenia occurred in 26.9% of patients and was reversible with conservative therapy. Substantial deterioration of general conditions owing to nausea or vomiting was a major gastrointestinal problem during chemoradiotherapy, and two patients did not complete the scheduled radiation due to gastrointestinal toxicity. Some reports showed fatal gastrointestinal toxicity, especially ulceration and bleeding of the duodenum (McGinn *et al*, 2001; Brunner *et al*, 2003). We observed one patient (2.4%) with duodenal ulcer bleeding after completion of chemoradotherapy who was treated conservatively. Five other patients (12.2%) with gastric or duodenal ulcer were also successfully managed. In the setting of chemoradiation, cisplain-induced neuropathy is not a major toxicity (Brunner *et al*, 2003; Haddock *et al*, 2007). We had only one patient (2.4%) with grade 2 neuropathy during chemoradiation. During maintenance therapy, grades 1–3 neuropathy occurred in 12.1% of patients.

In the present study, we demonstrated that performance status, baseline weight loss, baseline serum CA19-9 level, and objective therapeutic response were significant predictors of survival by univariate analysis. A recent study by Ferrone *et al* (2006) reported that preoperative CA19-9 levels could be used as predictors of survival in patients with resectable pancreatic cancer. Their work showed that the cutoff level of 1000 U ml^−1^ had a strong correlation with survival (Ferrone *et al*, 2006). We showed that patients with less than 1000 U ml^−1^ of baseline serum CA19-9 level were long-term survivors.

We performed PET scanning to evaluate distant metastasis in all enrolled patients. Our previous report demonstrated that initial stages using CT scan were changed in 26.9% patients with pancreatic cancer after PET scan (Bang *et al*, 2006a). Positron emission tomography scans showed high sensitivity and specificity in detecting metastatic disease, including liver, lungs, and peritoneum (Pakzad *et al*, 2006; Bang *et al*, 2006a). Initial staging workup, including PET scans, might be beneficial in selecting good candidates for chemoradiotherapy in locally advanced pancreatic cancer.

This study has a limitation of precise evaluation of resectability of primary pancreatic cancer after chemoradiotherapy. Computed tomography scans make it difficult to differentiate remnant tumours with radiation-induced change in re-staging after chemoradiotherapy. A previous study showed that SD re-staged by CT scan revealed pathological PR after laparotomy (Mornex *et al*, 2006).

In conclusion, we demonstrated the clinical efficacy and tolerability of treating locally advanced pancreatic cancer with a weekly full dose of gemcitabine and a single dose of cisplatin with concurrent radiotherapy. Combining chemotherapeutic agents are important to achieve not only local control but also good systemic cytotoxicity in patients with locally advanced pancreatic cancer.

## Figures and Tables

**Table 1:** Patient characteristics

**Variable**	**No. of patients (%)**
Enrolled patients	41
	
*Age (years)*	
Median (range)	59 (37–72)
	
*Sex*	
Male	22 (53.7)
Female	19 (46.3)
	
*Performance status*	
ECOG, 0–1	23 (56.1)
ECOG, 2	18 (43.9)
	
Diabetes mellitus	13 (31.7)
	
*Symptoms at baseline*	
Abdominal pain	31 (75.6)
Jaundice	15 (36.6)
Weight loss	22 (53.7)
	
*Tumour site*	
Head	26 (63.4)
Body	12 (29.3)
Tail	3 (7.3)
	
*Tumour size (longest diameter, cm)*	
Median (range)	3.2 (1.5–9.0)
	
*Tumour stage*	
IIA	8 (19.5)
IIB	3 (7.3)
III	30 (73.2)
	
CA 19-9 (U ml^−1^) increased	33 (80.5)
Median (range)	686 (51–20 000)

ECOG=Easter Cooperative Oncology Group.

**Table 2:** Parameters influencing cumulative survival of patients analysed by univariate analysis

**Parameters**	**Median survival time (months)**	**95% CI (months)**	***P*-value**
*Age*			0.572
<60	15.6	8.6–22.7	
⩾60	16.7	8.0–25.5	
			
*Sex*			0.167
Male	20.6	10.3–31.0	
Female	12.6	4.2–21.0	
			
*Performance status*			0.002
ECOG, 0–1	20.1	16.8–23.4	
ECOG, 2	10.8	6.0–15.5	
			
*Weight loss, baseline*			0.022
None	20.3	16.2–24.5	
1–5 kg	16.7	1.6–31.8	
6–10 kg	12.6	5.4–19.7	
>10 kg	6.0	0–13.8	
			
*Tumour site*			0.152
Head	20.2	13.8–26.7	
Body-tail	11.9	8.1–15.7	
			
*Tumour size*			
⩾5 cm	20.1	0.7–39.6	0.137
<5 cm	15.9	10.7–21.2	
			
*T stage*			0.556
T3	18.3	7.8–28.7	
T4	15.9	10.9–21.0	
			
*N* *stage*			0.992
N1	15.6	6.1–25.2	
N0	16.7	10.1–23.3	
			
*CA19-9, baseline*			0.004
<1000 U ml^−1^	20.1	16.2–24.1	
⩾1000 U ml^−1^	8.2	6.3–10.0	
			
*Objective response*			0.019
CR+PR	18.3	6.2–30.3	
SD	16.7	13.4–20.1	
PD	7.5	5.9–9.2	
			
*Surgery*			0.079
Yes	21.7	14.1–29.3	
No	12.6	7.5–17.7	

CR=complete response; ECOG=Easter Cooperative Oncology Group; PD=progressive disease; PR=partial response; SD=stable disease.

**Table 3:** Treatment-related toxicities according to WHO toxicity criteria

	**No. of patients (%)**			
**Toxicities**	**Grade 1**	**Grade 2**	**Grade 3**	**Grade 4**
*Haematology*				
Neutropaenia	9 (22.0)	11 (26.8)	7 (17.1)	4 (9.8)
Anaemia	12 (29.3)	24 (58.5)	0 (0)	0 (0)
Thrombocytopaenia	18 (43.9)	7 (17.1)	8 (19.5)	0 (0)
				
*Nonhaematology*				
Nausea/vomiting	7 (17.1)	7 (17.1)	9 (22.0)	0 (0)
Mucositis	5 (12.2)	2 (4.9)	0 (0)	0 (0)
Diarrhoea	1 (2.4)	2 (4.9)	2 (4.9)	0 (0)
Gastric ulcer	0 (0)	3 (7.3)	0 (0)	0 (0)
Duodenal ulcer	0 (0)	2 (4.9)	1 (2.4)	0 (0)
AST/ALT	3 (7.3)	6 (14.6)	0 (0)	0 (0)
Neuropathy	0 (0)	1 (2.4)	0 (0)	0 (0)
Hypersensitivity	3 (7.3)	1 (2.4)	0 (0)	0 (0)
Infection	1 (2.4)	2 (4.9)	1 (2.4)	0 (0)

ALT=alanine transaminase; AST=aspartate transaminase.

**Table 4:** Summary of phase II trials of chemoradiotherapy combined with gemcitabine alone or gemcitabine plus cisplatin in locally advanced pancreatic cancer

**Study**	**No. of patients**	**Chemotherapy dose (mg m^−2^)**	**RT dose (Gy)**	**CR+PR (%)**	**Median TTP (months)**	**Median OS (months)**	**1-year survival (%)**
de Lange *et al* (2002)	24	G 300 weekly per 3 weeks	24	29.2	7	10	—
Li *et al* (2003)	18	G 600 weekly per 6 weeks	50.4–61.2	50	7.1	14.5	—
Wilkowski *et al* (2004)	47	G 300, C 30 weekly per 4 weeks	45–50	68	7.8	10.7	—
Okusaka *et al* (2004)	42	G 250 weekly per 6 weeks	50.4	21	4.4	9.5	28
Magnino *et al* (2005)	23	G 50, 100 biweekly per 5 weeks	45	22	—	14	—
Haddock *et al* (2007)	48	G 30, C 10 biweekly per 3 weeks	50.4	8	7.3	10.2	40.4

C=cisplatin; CR=complete response; G=gemcitabine; OS=overall survival; PR=partial response; RT=radiotherapy; TTP=time to progression.

## References

[bib1] A'Hern RP (2001) Sample size tables for exact single-stage phase II designs. Stat Med 20: 859–866 11252008 10.1002/sim.721

[bib2] Ammori JB, Colletti LM, Zalupski MM, Eckhauser FE, Greenson JK, Dimick J, Lawrence TS, McGinn CJ (2003) Surgical resection following radiation therapy with concurrent gemcitabine in patients with previously unresectable adenocarcinoma of the pancreas. J Gastrointest Surg 7: 766–772 13129554 10.1016/s1091-255x(03)00113-6

[bib3] Bang S, Chung HW, Park SW, Chung JB, Yun M, Lee JD, Song SY (2006a) The clinical usefulness of 18-fluorodeoxyglucose positron emission tomography in the differential diagnosis, staging, and response evaluation after concurrent chemoradiotherapy for pancreatic cancer. J Clin Gastroenterol 40: 923–929 17063113 10.1097/01.mcg.0000225672.68852.05

[bib4] Bang S, Jeon TJ, Kim MH, Park JY, Park SW, Chung JB, Song SY (2006b) Phase II study of cisplatin combined with weekly gemcitabine in the treatment of patients with metastatic pancreatic carcinoma. Pancreatology 6: 635–641 17159377 10.1159/000097784

[bib5] Bergman AM, Ruiz van Haperen VW, Veerman G, Kuiper CM, Peters GJ (1996) Synergistic interaction between cisplatin and gemcitabine *in vitro*. Clin Cancer Res 2: 521–530 9816199

[bib6] Blackstock AW, Bernard SA, Richards F, Eagle KS, Case LD, Poole ME, Savage PD, Tepper JE (1999) Phase I trial of twice-weekly gemcitabine and concurrent radiation in patients with advanced pancreatic cancer. J Clin Oncol 17: 2208–2212 10561277 10.1200/JCO.1999.17.7.2208

[bib7] Brunner TB, Grabenbauer GG, Klein P, Baum U, Papadopoulos T, Bautz W, Hohenberger W, Sauer R (2003) Phase I trial of strictly time-scheduled gemcitabine and cisplatin with concurrent radiotherapy in patients with locally advanced pancreatic cancer. Int J Radiat Oncol Biol Phys 55: 144–153 12504047 10.1016/s0360-3016(02)03818-x

[bib8] Chung HW, Bang SM, Park SW, Chung JB, Kang JK, Kim JW, Seong JS, Lee WJ, Song SY (2004) A prospective randomized study of gemcitabine with doxifluridine versus paclitaxel with doxifluridine in concurrent chemoradiotherapy for locally advanced pancreatic cancer. Int J Radiat Oncol Biol Phys 60: 1494–1501 15590180 10.1016/j.ijrobp.2004.05.061

[bib9] Colucci G, Giuliani F, Gebbia V, Biglietto M, Rabitti P, Uomo G, Cigolari S, Testa A, Maiello E, Lopez M (2002) Gemcitabine alone or with cisplatin for the treatment of patients with locally advanced and/or metastatic pancreatic carcinoma: a prospective, randomized phase III study of the Gruppo Oncologia dell'Italia Meridionale. Cancer 94: 902–910 11920457

[bib10] de Lange SM, van Groeningen CJ, Meijer OW, Cuesta MA, Langendijk JA, van Riel JM, Pinedo HM, Peters GJ, Meijer S, Slotman BJ, Giaccone G (2002) Gemcitabine-radiotherapy in patients with locally advanced pancreatic cancer. Eur J Cancer 38: 1212–1217 12044508 10.1016/s0959-8049(02)00076-x

[bib11] Ferrone CR, Finkelstein DM, Thayer SP, Muzikansky A, Fernandez-delCastillo C, Warshaw AL (2006) Perioperative CA19-9 levels can predict stage and survival in patients with resectable pancreatic adenocarcinoma. J Clin Oncol 24: 2897–2902 16782929 10.1200/JCO.2005.05.3934 PMC3817569

[bib12] Gastrointestinal Tumor Study Group (1979) A multi-institutional comparative trial of radiation therapy alone and in combination with 5-fluorouracil for locally unresectable pancreatic carcinoma. Ann Surg 189: 205–208 426553 PMC1397020

[bib13] Gastrointestinal Tumor Study Group (1988) Treatment of locally unresectable carcinoma of the pancreas: comparison of combined-modality therapy (chemotherapy plus radiotherapy) to chemotherapy alone. J Natl Cancer Inst 80: 751–755 2898536

[bib14] Haddock MG, Swaminathan R, Foster NR, Hauge MD, Martenson JA, Camoriano JK, Stella PJ, Tenglin RC, Schaefer PL, Moore DF, Alberts SR (2007) Gemcitabine, cisplatin, and radiotherapy for patients with locally advanced pancreatic adenocarcinoma: results of the North central cancer treatment group phase II study N9942. J Clin Oncol 25: 2567–2572 17577035 10.1200/JCO.2006.10.2111

[bib15] Heinemann V, Quietzsch D, Gieseler F, Gonnermann M, Schönekäs H, Rost A, Neuhaus H, Haag C, Clemens M, Heinrich B, Vehling-Kaiser U, Fuchs M, Fleckenstein D, Gesierich W, Uthgenannt D, Einsele H, Holstege A, Hinke A, Schalhorn A, Wilkowski R (2006) Randomized phase III trial of gemcitabine plus cisplatin compared with gemcitabine alone in advanced pancreatic cancer. J Clin Oncol 24: 3946–3952 16921047 10.1200/JCO.2005.05.1490

[bib16] Jemal A, Murray T, Ward E, Samuels A, Tiwari RC, Ghafoor A, Feuer EJ, Thun MJ (2005) Cancer statistics, 2005. CA Cancer J Clin 55: 10–30 15661684 10.3322/canjclin.55.1.10

[bib17] Kalser MH, Barkin J, MacIntyre JM (1985) Pancreatic cancer. Assessment of prognosis by clinical presentation. Cancer 56: 397–402 4005804 10.1002/1097-0142(19850715)56:2<397::aid-cncr2820560232>3.0.co;2-i

[bib18] Li CP, Chao Y, Chi KH, Chan WK, Teng HC, Lee RC, Chang FY, Lee SD, Yen SH (2003) Concurrent chemoradiotherapy treatment of locally advanced pancreatic cancer: gemcitabine versus 5-fluorouracil, a randomized controlled study. Int J Radiat Oncol Biol Phys 57: 98–104 12909221 10.1016/s0360-3016(03)00435-8

[bib19] Magnino A, Gatti M, Massucco P, Sperti E, Faggiuolo R, Regge D, Capussotti L, Gabriele P, Aglietta M (2005) Phase II trial of primary radiation therapy and concurrent chemotherapy for patients with locally advanced pancreatic cancer. Oncology 68: 493–499 16020980 10.1159/000086993

[bib20] McGinn CJ, Zalupski MM, Shureiqi I, Robertson JM, Eckhauser FE, Smith DC, Brown D, Hejna G, Strawderman M, Normolle D, Lawrence TS (2001) Phase I trial of radiation dose escalation with concurrent weekly full-dose gemcitabine in patients with advanced pancreatic cancer. J Clin Oncol 19: 4202–4208 11709563 10.1200/JCO.2001.19.22.4202

[bib21] Ministry of Health and Welfare Republic of Korea (2007) Annual report of national cancer registration 1999–2002

[bib22] Moertel CG, Frytak S, Hahn RG, O'Connell MJ, Reitemeier RJ, Rubin J, Schutt AJ, Weiland LH, Childs DS, Holbrook MA, Lavin PT, Livstone E, Spiro H, Knowlton A, Kalser M, Barkin J, Lessner H, Mann-Kaplan R, Ramming K, Douglas HO, Thomas P, Nave H, Bateman J, Lokich J, Brooks J, Chaffey J, Corson JM, Zamcheck N, Novak JW (1981) Therapy of locally unresectable pancreatic carcinoma: a randomized comparison of high dose (6000 rads) radiation alone, moderate dose radiation (4000 rads + 5-fluorouracil), and high dose radiation + 5-fluorouracil: the gastrointestinal tumor study group. Cancer 48: 1705–1710 7284971 10.1002/1097-0142(19811015)48:8<1705::aid-cncr2820480803>3.0.co;2-4

[bib23] Mornex F, Girard N, Scoazec JY, Bossard N, Ychou M, Smith D, Seitz JF, Valette PJ, Roy P, Rouanet P, Ducreux M, Partensky C (2006) Feasibility of preoperative combined radiation therapy and chemotherapy with 5-fluorouracil and cisplatin in potentially resectable pancreatic adenocarcinoma: the French SFRO-FFCD 97-04 Phase II trial. Int J Radiat Oncol Biol Phys 65: 1471–1478 16793214 10.1016/j.ijrobp.2006.02.054

[bib24] Okusaka T, Ito Y, Ueno H, Ikeda M, Takezako Y, Morizane C, Kagami Y, Ikeda H (2004) Phase II study of radiotherapy combined with gemcitabine for locally advanced pancreatic cancer. Br J Cancer 91: 673–677 15226765 10.1038/sj.bjc.6602001 PMC2364779

[bib25] Pakzad F, Groves AM, Ell PJ (2006) The role of positron emission tomography in the management of pancreatic cancer. Semin Nucl Med 36: 248–256 16762614 10.1053/j.semnuclmed.2006.03.005

[bib26] Paulino AC, Latona C (2000) Unresectable adenocarcinoma of the pancreas: patterns of failure and treatment results. Cancer Invest 18: 309–313 10808366 10.3109/07357900009012173

[bib27] Peters GJ, Bergman AM, Ruiz van Haperen VW, Veerman G, Kuiper CM, Braakhuis BJ (1995) Interaction between cisplatin and gemcitabine *in vitro* and *in vivo*. Semin Oncol 22: 72–79 7481849

[bib28] Snady H, Bruckner H, Cooperman A, Paradiso J, Kiefer L (2000) Survival advantage of combined chemoradiotherapy compared with resection as the initial treatment of patients with regional pancreatic carcinoma. An outcomes trial. Cancer 89: 314–327 10918161 10.1002/1097-0142(20000715)89:2<314::aid-cncr16>3.0.co;2-v

[bib29] Wilkowski R, Thoma M, Schauer R, Wagner A, Heinemann V (2004) Effect of chemoradiotherapy with gemcitabine and cisplatin on locoregional control in patients with primary inoperable pancreatic cancer. World J Surg 28: 1011–1018 15573257 10.1007/s00268-004-7338-z

[bib30] World Health Organization (1979) WHO Handbook for Reporting Results of Cancer Treatment Offset Publication 48. Geneva: World Health Organization

[bib31] Yeo CJ, Cameron JL, Sohn TA, Lillemoe KD, Pitt HA, Talamini MA, Hruban RH, Ord SE, Sauter PK, Coleman J, Zahurak ML, Grochow LB, Abrams RA (1997) Six hundred fifty consecutive pancreaticoduodenectomies in the 1990s: pathology, complications, and outcomes. Ann Surg 226: 248–257; discussion 257 9339931 10.1097/00000658-199709000-00004 PMC1191017

